# A structured exercise to relieve musculoskeletal pain caused by face-down posture after retinal surgery: a randomized controlled trial

**DOI:** 10.1038/s41598-021-01182-w

**Published:** 2021-11-11

**Authors:** A-Young Kim, Sungsoon Hwang, Se Woong Kang, So Yeon Shin, Won Hyuk Chang, Sang Jin Kim, Hoon Noh

**Affiliations:** 1 Department of Ophthalmology, Samsung Medical Center, Sungkyunkwan University School of Medicine, #81 Irwon-ro, Gangnam-gu, Seoul, 06351 Republic of Korea; 2 Department of Ophthalmology, Ewha Womans University Seoul Hospital, Ewha Womans University School of Medicine, Seoul, Republic of Korea; 3 Department of Clinical Research Design and Evaluation, Samsung Advanced Institute for Health Sciences and Technology (SAIHST), Sungkyunkwan University, Seoul, Republic of Korea; 4 Department of Nursing, Samsung Medical Center, Seoul, Republic of Korea; 5 Department of Physical and Rehabilitation Medicine, Center for Prevention and Rehabilitation, Heart Vascular Stroke Institute, Samsung Medical Center, Sungkyunkwan University School of Medicine, Seoul, Republic of Korea

**Keywords:** Retinal diseases, Randomized controlled trials

## Abstract

Face-down posture after vitrectomy physically burdens patients. Despite being of significant concern for patients, the intraoperative pain and discomfort has not been of great interest to retinal surgeons or researchers. This randomized controlled trial evaluated the effect of a 3-day novel structured exercise on reducing musculoskeletal pain from the face-down posture in 61 participants (31 in the exercise group) who underwent vitrectomy. Among the subjects, the median age was 62 years, 42 were female, 42 had macular holes, and 19 had retinal detachments. Participants in the exercise group received initial education on the exercise and performed three daily active exercise sessions. After the sessions, the exercise group had median numeric pain scores of 2, 1, and 1 at the back neck, shoulder, and lower back, respectively, while the control group had corresponding scores of 5, 3, and 4, respectively. The exercise group reported significantly lower pain scores (*P* = .003, .039, and .006 for the back neck, shoulder, and lower back, respectively). Application of the structured exercise would alleviate the patients’ position-induced postoperative physical burden, by reducing pain and discomfort.

## Introduction

Face-down positioning (FDP) is a standard posture recommended after vitrectomy and intraocular tamponade for macular holes and retinal detachments. With postoperative FDP, intraocular gas or oil mechanically pushes the retina against the retinal pigment epithelium, keeping the sensory retina attached, and isolating a retinal tear or macular hole from the vitreous fluid^1^. Its appropriate duration remains controversial; however, it is generally advised to maintain FDP for ≥ 3 days, depending on the retinal condition.

Postoperative FDP and immobilization cause considerable physical and psychological burden to patients^2^. Patients positioned face-down complain of neck, shoulder, and back pain, as well as mental stress, a sense of psychological isolation, and anxiety^2^. Some also experience pressure ulcers and ulnar neuropathies^3,4^. Associated pain and discomfort may thus lead to reduced compliance. Seno et al.^5^ reported that FDP maintenance after vitrectomy and gas tamponade varied considerably among patients. The reduced proper posture holding time caused by discomfort from the posture could decrease its therapeutic effect, thereby negatively affecting the clinical outcome of the surgery.

FDP-related musculoskeletal pain has not been a major concern for researchers, despite being of substantial concern for patients. Limited studies have quantitatively investigated FDP-related musculoskeletal pain, and little effort has been made to reduce the FDP-related pain. To our knowledge, no previous study has suggested or evaluated a proper posture and structured rehabilitation exercise for reducing FDP-related pain. We hypothesized that application of the structured exercise in patients on FDP would lessen their physical stress and improve postoperative satisfaction. In this study, we devised a novel structured rehabilitation exercise program and validated the effects of exercise on FDP-related musculoskeletal pain after retinal surgery for macular holes and retinal detachments.

## Methods

### Study design, setting, sample size

**Figure 1:**
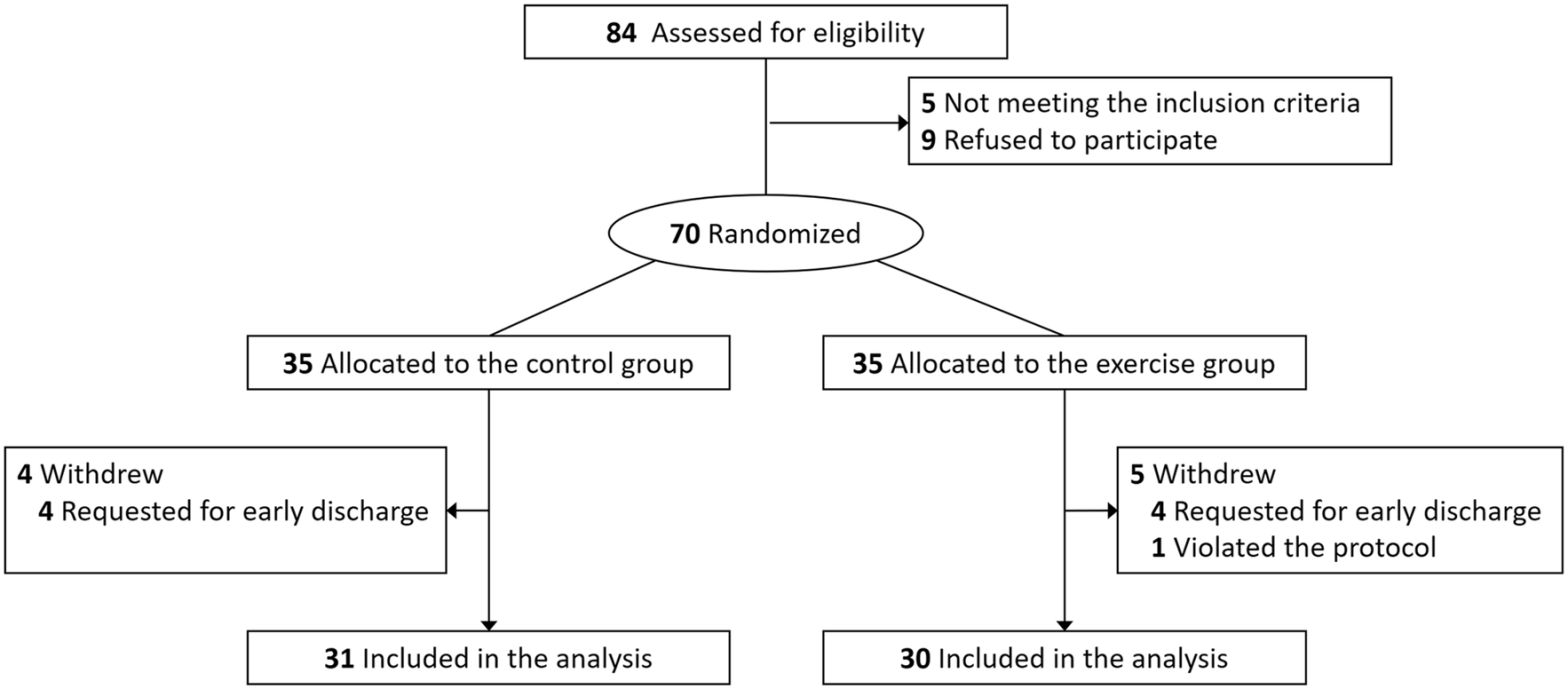
CONSORT flow diagram showing participant screening, recruitment, and randomization.

This prospective, single-masked, randomized clinical trial was performed at the Samsung Medical Center, Seoul from September 3 to December 14, 2020. It was approved by the institutional review board of Samsung Medical Center (no. 2019-03-157), was registered at ClinicalTrials.gov (identifier: NCT04535622), and adhered to the tenets of the Declaration of Helsinki. Written informed consent was obtained from all participants. A total of 70 participants were enrolled in the study and the process of recruitment and allocation is described in Fig. 1 (Consolidated Standards of Reporting Trials flow diagram). The formal trial protocols and statistical analysis design are shown in the Supplemental Document.

### Participants

The study included participants aged ≥ 18 years diagnosed with retinal detachment (both rhegmatogenous and tractional) or macular hole and admitted to the hospital for vitrectomy. Patients aged ≥ 80 years, with preexisting musculoskeletal disorders or poor best-corrected visual acuity in the opposite eye (less than 20/200 in the Snellen chart), which would make it difficult for them to follow instructions and participate, or those not scheduled for FDP and who decided to maintain sitting position postoperatively (e.g., pseudophakic patients with superior retinal detachment) were excluded. All participants were admitted to the hospital 1 day preoperatively and were randomized into either the exercise or control groups. They underwent vitrectomy performed by a surgeon (S.W.K.) who was blinded to the group allocation. A structured rehabilitation exercise program was applied to the exercise group. Exercise education and training were conducted by a physiotherapist affiliated with the Department of Physical and Rehabilitation Medicine after vitrectomy and the pain score was assessed. All other procedures and management were the same in both groups. Conventional painkillers, nonsteroidal anti-inflammatory drug skin patches, and positioning pillows were provided to both groups on a pro re nata basis. The patients were followed-up until postoperative day 3 and then discharged. The detailed hospital course of the two study groups is illustrated in Supplemental Fig. 1.

### Intervention

**Figure 2:**
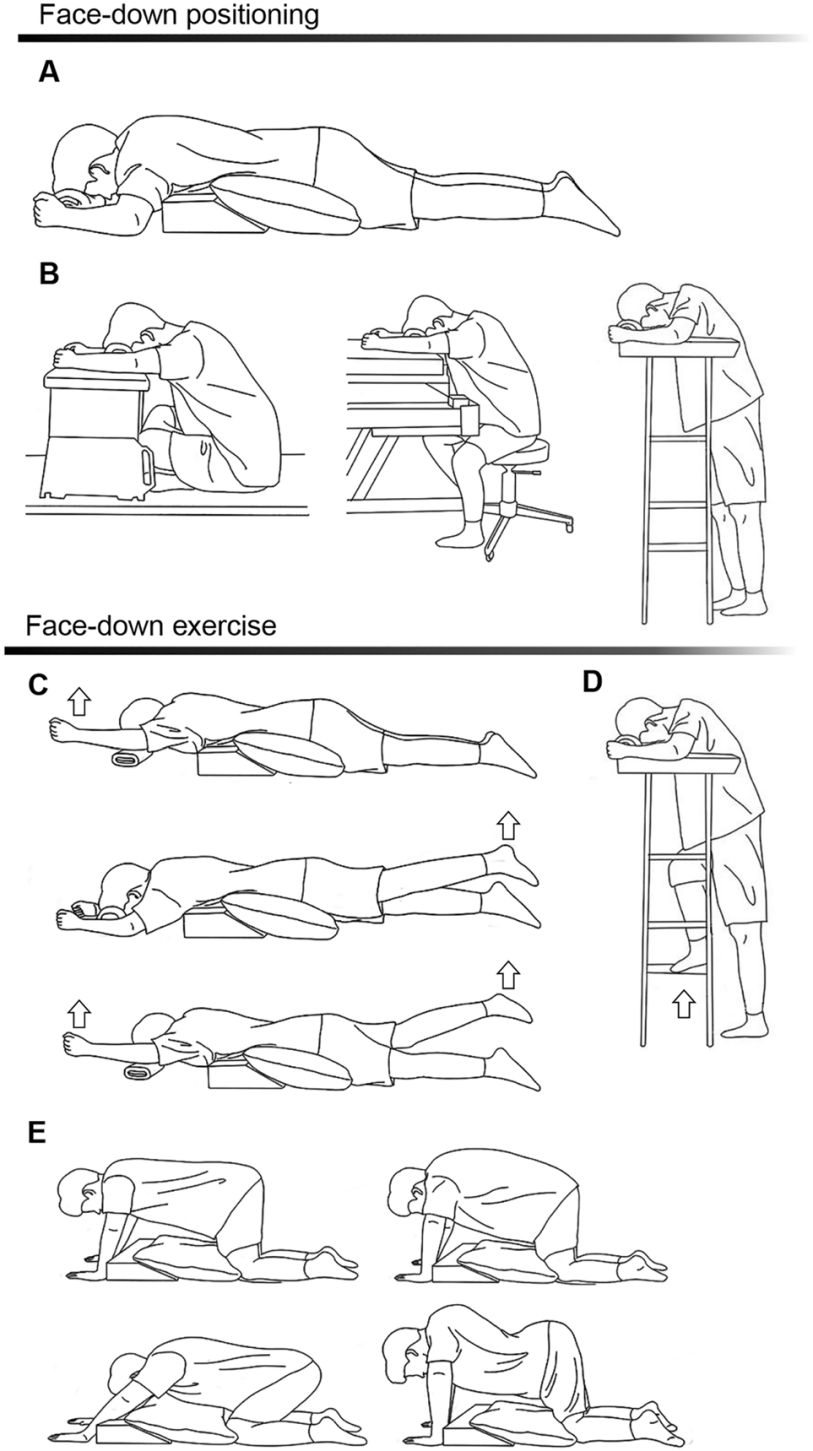
Patient Instructions for Proper FDP and Rehabilitation Exercise. (**A**) Basic FDP. Use a towel or pillow to support the forehead and reduce the load on the back neck. Use a pillow to support the upper body from the chest to the pelvis and keep them higher than the legs to reduce lower back load. Adjust pillow positioning to support the forehead and abdomen. (**B**) Modified FDP using a table (left), chair (middle), and walker (right). Maintaining only the basic FDP could put much strain on the neck, shoulders, and waist. Therefore, while maintaining basic FDP, modified FDP was occasionally applied, so that the physical stress is not concentrated in specific areas of the body. The following instructions were provided: (Left) Put a small table on the bed, and place the head on it. Use a towel or pillow to support your forehead, and place your arms naturally on either side of your head. (Middle) Adjust the height of the bed, place the chair at bedside, and maintain FDP while sitting. (Right) Adjust the height of the walking aids, and assume FDP in a standing position. Adjust the height of the bed, table, or walker properly, and use a pillow or towel under the forehead to avoid excessive neck or back bending. (**C**) Limb exercise in the basic FDP. Slowly lift one arm to shoulder level for 5–10 s, before putting it back into place. Slowly lift one leg to the level of the buttock with your straightened for 5 to 10 s, before put it back into place, to avoid straining the waist. The postures are then combined, such that the arms and legs are crossed. Each posture was maintained for 5 to 10 s and performed 10 times twice. This exercise stretches and strengthens the limbs and lower back muscles. (**D**) Limb exercise in standing FDP with walking aids. Walk slowly, and try not to lose balance and fall down. Each side was repeated 10 times for two to three sets. This exercise strengthens the muscles in the back and lower extremities. (**E**) Exercise in the hand and knee position: back stretching (left two rows), back bending, and forward bending (right two rows). With the head face down, take the hand-and-knee position. Sit back with the hips touching the heels with head maintained. Feel the stretch on your arms, shoulders, back, and buttocks; hold the position for 10–15 s; and then return to the hand-and-knee position. Repeat this for 5 to 10 times. Slowly lift the waist, making the spine round like a dome, then slower lower it, making the spine U-shaped. Maintain each posture for 5 to 10 s and performed 10 times twice. This exercise relaxes the shoulders, back, and spine, and strengthens the back muscles. The entire exercise session was repeated three times daily. During exercise, the position of the head should be maintained with the floor. This figure is created by Hani Yun from the Samsung Medical Information & Media Services, Samsung Medical Center.

The exercise education program was designed through a collaboration between the Department of Ophthalmology and the Department of Physical and Rehabilitation Medicine at the Samsung Medical Center to reduce muscular fatigue at the neck, shoulder, and waist, and to strengthen the muscles and joints while performing FDP. It comprised education on the following: (1) performing proper FDP without putting too much physical stress, and (2) exercise methods while maintaining FDP. The detailed methods are illustrated in Fig. 2 and available online as a movie clip with English subtitles^6^. The exercises were performed within the range of each individual muscle’s strength and fitness. The patients were asked to maintain proper FDP and perform self-exercise sessions three times daily. The physical therapist provided feedback to the participants on the next day of education.

### Comparison

The randomization process was implemented by an independent clinical trial consultant. The patients were randomized in a 1:1 ratio using a block randomization method with permutated blocks of 4 or 6 in size. Subjects were allocated blocks based on pre-allocated codes placed in sealed opaque envelopes that were opened by a trial coordinator during the randomization step. Based on the code, each subject was randomized to either the control or the exercise group. Owing to the nature of the intervention, the participants, healthcare professionals, and researchers could not be blinded to the group allocation. Only the data manager, trial statisticians, and surgeon at the time of surgery were masked to the allocation.

### Endpoint

The musculoskeletal pain scores at the back neck, shoulder, and lower back were assessed immediately after the surgery and on postoperative days 1, 2, and 3. The pain score was assessed using the numeric rating scale (NRS) scoring system, wherein the pain level was quantified on a scale of 0 (no pain) to 10 (worst possible pain). The NRS is a valid and frequently employed method of musculoskeletal pain assessment, and its high validity and reliability have been reported by many studies^7^. The primary endpoints were musculoskeletal pain scores at the back neck, shoulder, and lower back on postoperative day 3. The secondary endpoints included musculoskeletal pain scores at the back neck, shoulder, and lower back on postoperative days 0, 1, and 2. Additionally, a satisfaction survey on the exercise program was conducted for the exercise group on postoperative day 3. The questionnaire included items regarding whether the exercise program helped the patients’ postoperative recovery, whether the patients would like to receive this program again in case of re-operation, whether they would recommend the program to other patients, and their overall satisfaction level (Supplemental Fig. 2).

### Statistical analysis

No relevant studies have previously described the effect of structured exercise on FDP-related pain. One previous study performed complementary therapy and quantitatively evaluated FDP-related pain^8^. Based on the clinical assumption that structured exercise programs would have effects comparable to or greater than those obtained via complementary therapy on FDP-related pain, we conducted a sample size calculation expecting an effect size of 0.9 based on post-treatment pain scores in the aromatherapy and control groups of the previous study^8^. The estimated sample size, power, and Bonferroni-corrected significance level were 32 patients, 90%, and 0.05, respectively. Considering a 10% loss to follow-up, we recruited 35 patients in each arm.

Statistical analysis was performed using SPSS (version 23.0; SPSS, Inc., Chicago, IL, USA). The Chi-square test and Wilcoxon rank-sum test were used to analyze the baseline characteristics of both groups. The Wilcoxon rank-sum test was used to compare the musculoskeletal pain scores. Bonferroni’s correction for multiple testing was applied to compare the pain scores assessed at three different sites (back neck, shoulder, and lower back). *P*-values of < 0.05 were considered statistically significant.

## Results

### Baseline characteristics

A total of 70 participants were enrolled (Fig. 1). Eight subjects requested study withdrawal and early discharge, and one more refused to undergo the structured exercise program. Therefore, 61 participants were included in the analysis. Their median age was 62 years (interquartile range, 53–69 years). Among the participants, 42 (68.9%) were women, 42 (68.9%) were diagnosed with macular holes, and 19 (31.1%) had retinal detachments. The baseline characteristics of the two groups were similar and are summarized in Table 1.

**Table 1:** Baseline characteristics of the study participants.

	Control group	Exercise group	*P*-value
Participants, no	31	30	
Age, years, median (IQR)	62 (51–70)	59.5 (55–66)	.750
**Sex, no. (%)**			1.000
Male	10 (32.3)	9 (30.0)	
Female	21 (67.7)	21 (70.0)	
Diabetes mellitus, no. (%)	6 (19.4)	2 (6.7)	.255
Hypertension, no. (%)	11 (35.5)	9 (30.0)	.786
**Indication for surgery, no. (%)**			.786
Macular hole	22 (71.0)	20 (66.7)	
Retinal detachment	9 (29.0)	10 (33.3)	
**Laterality, no. (%)**			1.000
Right	16 (51.6)	16 (53.3)	
Left	15 (48.4)	14 (46.7)	
Previous experience in retinal surgeries with face-down positioning, no. (%)	3 (9.7)	2 (6.7)	1.000
Combined cataract surgery, no. (%)	20 (64.5)	20 (66.7)	1.000
**Filling material, no. (%)**			1.000
SF6	16 (48.4)	15 (50.0)	
C3F8	13 (45.2)	12 (40.0)	
Silicone oil	2 (6.5)	3 (10.0)	
**Anesthesia, no. (%)**			.707
Local	26 (83.9)	27 (90.0)	
General	5 (16.1)	3 (10.0)	
Surgery time, minutes, median (IQR)	53 (45–66)	57 (43–71)	.526

The Mann–Whitney test and Fisher’s exact test were used for continuous and categorical variables, respectively.

IQR, interquartile range; SF6, sulfur hexafluoride; C3F8, octafluoropropane.

### Outcomes

The NRS pain scores in the study groups are shown in Table 2. On postoperative day 3, the exercise group had median pain scores of 2, 1, and 1 at the back neck, shoulder, and lower back, respectively, while the control group had corresponding scores of 5, 3, and 4, respectively. The exercise group had significantly lower back neck, shoulder, and lower back pain scores (*P* = 0.003, 0.012, and 0.012, respectively). Conversely, the pain scores on postoperative days 0, 1, and 2 did not significantly differ between the two groups.

**Table 2:** Musculoskeletal pain scores of the study groups.

Postoperative day	Site	NRS Pain Score, Median (IQR)		*P*-value^a^
		Control group (n = 31)	Exercise group (n = 30)	
Day 0	Back neck	0.00 (0.00–1.00)	0.00 (0.00–0.00)	.259
	Shoulder	0.00 (0.00–1.00)	0.00 (0.00–1.25)	.871
	Lower back	0.00 (0.00–3.00)	0.00 (0.00–1.25)	.329
Day 1	Back neck	2.00 (0.00–5.00)	3.00 (2.00–5.00)	.275
	Shoulder	2.00 (0.00–5.00)	2.00 (0.00–4.25)	.904
	Lower back	3.00 (0.00–6.00)	2.50 (1.00–5.00)	.963
Day 2	Back neck	4.00 (2.00–6.00)	3.00 (1.00–5.00)	.345
	Shoulder	3.00 (2.00–5.00)	2.00 (1.00–4.00)	.146
	Lower back	2.00 (1.00–5.00)	1.00 (0.00–4.00)	.062
Day 3	Back neck	5.00 (3.00–5.00)	2.00 (0.75–3.25)	.001
	Shoulder	3.00 (2.00–5.00)	1.00 (0.00–4.00)	.013
	Lower back	4.00 (2.00–5.00)	1.00 (0.00–3.00)	.004

NRS, numeric rating scale; IQR, interquartile range.

^a^Bonferroni-corrected *P* values were obtained using the Mann–Whitney test.

**Figure 3:**
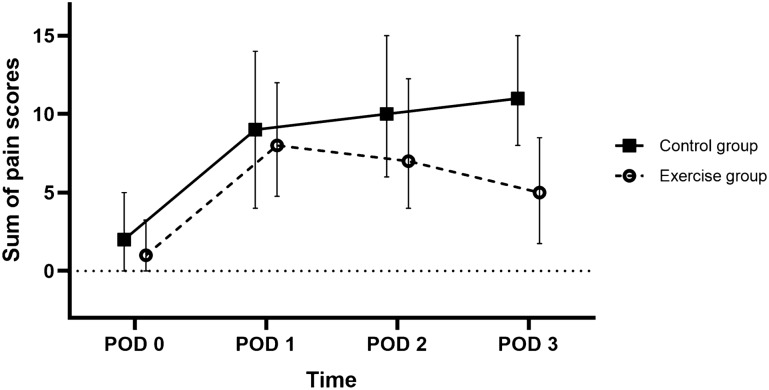
Time trend of the overall pain scores in the exercise and control groups. The data are shown as medians with interquartile ranges (error bars). The median pain scores gradually increased in the control group. Meanwhile, the median pain score increased on postoperative day (POD) 1 and then declined on POD 2–3 in the exercise group. The median total pain score (interquartile range) on POD 3 was 11 (8–15) and 5 (2–9) in the control and exercise groups, respectively.

Figure 3 demonstrates the changes in the overall NRS pain score over time. The overall pain score was calculated as the sum of the pain scores in the back neck, shoulder, and lower back to demonstrate the trend of overall physical burden of FDP. The overall pain score in the control group gradually increased until postoperative day 3, while that in the exercise group increased only on postoperative day 1 and started to decrease on the next day.

According to the satisfaction survey at discharge, 26 (87%) of the 30 subjects in the exercise group were satisfied with the exercise program. Detailed data on the satisfaction questionnaire are shown in Supplemental Fig. 2.

### Adverse events and anatomical outcomes

No associated complications or complaints were noted. At the 1-month follow-up, all participants presented successful anatomical outcomes (complete retinal reattachment or complete macular hole closure, *P* = 1.000 by Fisher’s exact test) and no musculoskeletal pain, except for three patients with retinal detachment who were lost to follow-up. No participants complained of musculoskeletal pain at the 1-month follow-up.

## Discussion

FDP causes significant discomfort to patients, and many efforts have been made to minimize the duration of FDP maintenance, or avoid strict prone posture after vitrectomy for retinal detachments and macular holes. Several studies have compared a strict FDP with other alternative postures. Chen et al.^9^ reported that adjustable positioning after vitrectomy and gas tamponade for rhegmatogenous retinal detachment repair was also effective. Other studies have suggested that alleviated positioning (avoidance of supine positioning) may have successful closure rates equivalent with those of FDP in macular hole surgery^10^. Other attempts have been made to reduce the posture-holding time, including the use of long-acting and short-acting gases^11–13^. Additionally, there have been some trials on adjusting the position or modifying the FDP period using optical coherence tomographic images in the gas-filled eye^14–16^. Attempts have also been made to compare the effects of gas and silicone oils^17–19^. However, meta-analyses comparing the clinical outcomes between postoperative FDP and non-FDP after macular hole surgery consistently indicated that FDP was necessary, especially for holes > 400 μm^20,21^. Regarding retinal detachment, a recent multicenter clinical trial suggested that compared with support-the-break positioning, FDP was associated with reduced rates of macular displacement and binocular diplopia after macula-involving retinal detachment repair^22^. Furthermore, strict FDP is still needed to avoid cataract and glaucoma development in gas-filled eyes^23–26^. In summary, postoperative FDP is still valid and necessary for improving surgical outcomes in many vitreous surgeries. Therefore, efforts are needed on understanding the discomfort caused by FDP, and reducing this physical burden.

Although FDP causes serious physical burden, pain, and mental isolation in patients, very few methods to counteract the posture-related discomfort have been reported. The introduced strategies involve the use of assistive devices, such as pillows, desks, and beds^27,28^, which are now generally implemented in the clinical setting. Passive interventions, such as painkillers and nonsteroidal anti-inflammatory drug patches, can also assist patients in maintaining FDP. Some clinics provide patients with brief instructions on the body posture to reduce discomfort, increase satisfaction, and motivate patients to exercise. However, these instructions are relatively informal and not systematically standardized. Hence, our study is important in that it proposed a structured instruction for proper posture and exercise and validated its efficacy for the first time.

In our study, the final back neck, shoulder, and lower back pain scores were significantly lower in those who received exercise education and performed regular structured exercise. In contrast, the pain scores on postoperative days 0, 1, and 2 did not significantly differ between the two groups. In the control group, the overall pain score continued to rise until the third day after surgery, whereas in the exercise group, the total pain score increased only on the first day after surgery and decreased continuously for the next 2 days. This result may indicate that exercise does not immediately reduce FDP-related physical pain, but has a long-term pain-relieving effect, emphasizing the importance of prolonged regular exercise. This is an encouraging result, considering that many patients need to maintain FDP for over 3 postoperative days. Unfortunately, we only observed the pain scores up to 3 days after surgery and were unable to assess the subsequent physical burden to the participants. However, the trend of the pain scores from postoperative day 1 to day 3 strongly suggests a benefit of structured exercise on FDP-related pain even after discharge. Questions may arise as to whether the reduced pain is attributable to the effects of the exercise itself or to the additional attention provided by the physical therapist. However, the physical therapist visited the patients once immediately after surgery and once for feedback on the first day after surgery, and it was on the third day after surgery that the two groups began to exhibit significant pain differences. If attention caused the improvement, the pain scores on the first or second postoperative days would also have significantly differed between the groups. Therefore, we believe that the structured exercise is mainly attributable to the alleviation of physical burden in the exercise group.

The reduction in musculoskeletal pain, which was our primary goal, was reaffirmed by evaluating patient satisfaction. Most patients were highly satisfied with the exercise program and were willing to participate in it again if they had to receive vitrectomy with postoperative FDP. They also responded that they would recommend this program to other patients. Therefore, this exercise program serves as a good example of promoting cooperation among different medical departments in the age of fusional medical care.

At the 1-month follow-up visit, all participants exhibited successful anatomical outcomes and did not complain of further musculoskeletal pain. Since the present study was initially designed to evaluate the influence of the structured exercise program on patient’s physical pain, its sample size might not have been sufficient to achieve enough statistical power for comparing the anatomical outcomes and long-term musculoskeletal effects of the positioning in the two groups. Additionally, even without significant influence on anatomical outcomes, structured exercise for FDP-related pain is still necessary. Not only the surgical outcomes, but also postoperative physical/psychological burdens are major concerns of patients undergoing retinal surgery^29,30^. Although the schedule for FDP is generally 3–10 days, the maintenance of the posture itself is physically challenging and may induce significant pain, fatigue, and physiological/psychological responses^31^. Therefore, reduction of physical burden is important for the patients’ experience, and the present study provides an effective method to achieve this goal.

The strength of our study is that it is the first to introduce a structured exercise for FDP-related musculoskeletal pain and to report its efficacy. We provided detailed instructions of the exercise sessions for widespread application. Our study also has several limitations. First, we analyzed the results after a relatively short period of FDP. Second, it was a single-center study, and the patient population was limited to those with macular holes and retinal detachments. Thus, caution is needed before generalizing the results to other retinal disorders or extended periods. However, the difference in pain scores between the two groups gradually widened, strongly suggesting a benefit of the exercise on musculoskeletal pain even after 3 days. Finally, this study was conducted in an inpatient hospital setting, raising the concern about the program’s utility in an outpatient-based vitrectomy setting. However, even at the hospital, the overall exercise was conducted by the patients themselves, following the initial education. The same initial education can be provided via brochures or as multimedia in an outpatient-based setting, and similar beneficial effects may be achieved if the provided instructions are followed.

In conclusion, the newly developed exercise program effectively reduced FDP-related musculoskeletal pain and yielded high patient satisfaction. A proper exercise program may increase patients’ compliance to FDP, thereby improving the surgical outcomes in some complex vitreous surgery cases. Moreover, the physical and psychological burden caused by FDP are important components of patient management, and greater attention should be paid to the patients’ experience in future research regarding retinal surgery.

## Supplementary Information

Supplementary Information 1.

Supplementary Information 2.

Supplementary Information 3.

## Data Availability

The datasets generated during and/or analyzed during the current study are available from the corresponding author on reasonable request.

## References

[1] Kelly NE, Wendel RT (1991). Vitreous surgery for idiopathic macular holes. Results of a pilot study (Chicago, Ill.: 1960). Arch. Ophthalmol..

[2] Harker R, McLauchlan R, MacDonald H, Waterman C, Waterman H (1996). Endless nights: Patients’ experiences of posturing face-down following vitreoretinal surgery. Ophthalmic Nurs..

[3] Treister G, Wygnanski T (1996). Pressure sore in a patient who underwent repair of a retinal tear with gas injection. Graefe's Arch. Clin. Exp. Ophthalmol..

[4] Brouzas D (2011). Ulnar neuropathy as a complication of retinal detachment surgery and face-down positioning. Case Rep. Ophthalmol..

[5] Seno Y, Shimada Y, Mizuguchi T, Tanikawa A, Horiguchi M (2015). Compliance with the face-down positioning after vitrectomy and gas tamponade for rhegmatogenous retinal detachments. Retina (Philadelphia, Pa.).

[6] Rehabilitation Exercise After Retinal Surgery [Video]. YouTube. https://www.youtube.com/watch?v=71XPgHp_0tc. Published August 4, 2020. Accessed December 14, 2020.

[7] Ferreira-Valente MA, Pais-Ribeiro JL, Jensen MP (2011). Validity of four pain intensity rating scales. Pain.

[8] Adachi N (2014). Effects of aromatherapy massage on face-down posture-related pain after vitrectomy: A randomized controlled trial. Pain Manag Nurs.

[9] Chen X, Yan Y, Hong L, Zhu L (2015). A comparison of strict face-down positioning with adjustable positioning after pars plana vitrectomy and gas tamponade for rhegmatogenous retinal detachment. Retina (Philadelphia, Pa.).

[10] Feist RM (2014). Nonsupine positioning is preferred by patients over face-down positioning and provides an equivalent closure rate in 25- and 23-gauge macular hole surgery. Retinal Cases Brief Rep..

[11] Essex RW (2016). The effect of postoperative face-down positioning and of long- versus short-acting gas in macular hole surgery: Results of a registry-based study. Ophthalmology.

[12] Iezzi R, Kapoor KG (2013). No face-down positioning and broad internal limiting membrane peeling in the surgical repair of idiopathic macular holes. Ophthalmology.

[13] Modi A, Giridhar A, Gopalakrishnan M (2017). Sulfurhexafluoride (SF6) versus perfluoropropane (C3F8) gas as tamponade in macular hole surgery. Retina (Philadelphia, Pa.).

[14] Kikushima W (2015). Dynamics of macular hole closure in gas-filled eyes within 24 h of surgery observed with swept source optical coherence tomography. Ophthalmic Res..

[15] Masuyama K (2009). Posturing time after macular hole surgery modified by optical coherence tomography images: A pilot study. Am. J. Ophthalmol..

[16] Yamashita T (2014). Individualized, spectral domain-optical coherence tomography-guided facedown posturing after macular hole surgery: Minimizing treatment burden and maximizing outcome. Retina (Philadelphia, Pa.).

[17] Banerjee PJ, Chandra A, Petrou P, Charteris DG (2017). Silicone oil versus gas tamponade for giant retinal tear-associated fovea-sparing retinal detachment: A comparison of outcome. Eye (Lond.).

[18] Bor'i A, Al-Aswad MA, Saad AA, Hamada D, Mahrous A (2017). Pars plana vitrectomy with internal limiting membrane peeling in traumatic macular hole: 14% perfluoropropane (C3F8) versus silicone oil tamponade. J. Ophthalmol..

[19] Lai JC, Stinnett SS, McCuen BW (2003). Comparison of silicone oil versus gas tamponade in the treatment of idiopathic full-thickness macular hole. Ophthalmology.

[20] Hu Z (2016). Face-down or no face-down posturing following macular hole surgery: A meta-analysis. Acta Ophthalmol..

[21] Xia S, Zhao XY, Wang EQ, Chen YX (2019). Comparison of face-down posturing with nonsupine posturing after macular hole surgery: A meta-analysis. BMC Ophthalmol..

[22] Casswell EJ (2020). Effect of face-down positioning vs support-the-break positioning after macula-involving retinal detachment repair: The PostRD randomized clinical trial. JAMA Ophthalmol..

[23] Chen, C. J. Glaucoma after macular hole surgery. *Ophthalmology* **105**, 94–99; discussion 99–100 (1998). 10.1016/s0161-6420(98)91470-1 9442784

[24] Kanclerz P, Grzybowski A (2018). Complications associated with the use of expandable gases in vitrectomy. J. Ophthalmol..

[25] Mangouritsas G (2013). Glaucoma associated with the management of rhegmatogenous retinal detachment. Clin. Ophthalmol. (Auckland, N.Z.).

[26] Schaefer H (2015). Can postoperative accelerated lens opacification be limited by lying in "face-down position" after vitrectomy with gas as tamponade?. Klin. Monatsbl. Augenheilkd..

[27] Rodanant N, Thompson C, Freeman WR (2002). A compact, inexpensive face-down positioning device. Retina (Philadelphia, Pa.).

[28] Hara T (2007). Total care system for maintaining a comfortable prone position after vitreoretinal surgery. Ophthalmic Surg. Lasers Imaging.

[29] Ellis JD, Baines PS (2002). Patient perspectives on macular hole surgery. Ophthalmology.

[30] Wittich W, Southall K (2008). Coping with extended facedown positioning after macular hole surgery: A qualitative diary analysis. Nurs. Res..

[31] Furushima C (2019). Influence of maintenance of face-down positioning on physiological and psychological factors. Vasc. Fail..

