# Supplementary material for: A structured exercise to relieve musculoskeletal pain caused by face-down posture after retinal surgery: a randomized controlled trial
Source: Sci Rep. 2021 Nov 11;11:22074. doi: 10.1038/s41598-021-01182-w (PMC8586155; doi:10.1038/s41598-021-01182-w)
Supplement: Supplementary file 1 — Supplementary Information 1. [file 41598_2021_1182_MOESM1_ESM.pdf]

A Structured Exercise to Relieve Musculoskeletal Pain Caused by Face-down Posture after Retinal Surgery: A Randomized Controlled Trial

A Young Kim, MD[1,2,*]; Sungsoon Hwang, MD[1,3,*]; Se Woong Kang, MD, PhD[1]; So Yeon Shin, RN[4]; Won Hyuk Chang, MD, PhD[5]; Sang Jin Kim, MD, PhD[1]; and Hoon Noh, MD[1]

[*]A Young Kim and Sungsoon Hwang contributed equally to this work as co-first authors.

[1]Department of Ophthalmology, Samsung Medical Center, Sungkyunkwan University School of Medicine, Seoul, Republic of Korea

[2]Department of Ophthalmology, Ewha Womans University Seoul Hospital, Ewha Womans University School of Medicine, Seoul, Republic of Korea

[3]Department of Clinical Research Design and Evaluation, Samsung Advanced Institute for Health Sciences and Technology (SAIHST), Sungkyunkwan University, Seoul, Republic of Korea

[4]Department of Nursing, Samsung Medical Center, Seoul, Republic of Korea

[5]Department of Physical and Rehabilitation Medicine, Center for Prevention and Rehabilitation, Heart Vascular Stroke Institute, Samsung Medical Center, Sungkyunkwan University School of Medicine, Seoul, Republic of Korea

Corresponding Author:

Se Woong Kang, MD, PhD

Department of Ophthalmology, Samsung Medical Center, Sungkyunkwan University School of Medicine, #81 Irwon-ro, Gangnam-gu, Seoul 06351, Republic of Korea

Tel: +82-2-3410-3562, Fax: +82-2-3410-0074

Email: kangsewoong@gmail.com

Clinical Trial Protocol

1. Full Title of the Clinical Trial

A randomized controlled trial to evaluate effect of a structured rehabilitation exercise on facedown posture-related musculoskeletal pain after retinal surgery

2. Sites and Address

A single center study: Samsung Medical Center, Sungkyunkwan University School of Medicine, 81 Irwon-ro, Gangnam-gu, Seoul, 06351, Korea

3. Investigators and Affiliation

Principal Investigator: Se Woong Kang, MD, PhD[1]

Co-investigators: Won Hyuk Chang, MD, PhD[2], A Young Kim, MD[1], Sang Jin Kim, MD, PhD[1], Hoon Noh, MD[1], Sungsoon Hwang, MD[1], So Yeon Shin, RN[3]

[1]Department of Ophthalmology, Samsung Medical Center, Sungkyunkwan University School of Medicine, Seoul, Korea

[2]Department of Ophthalmology, Ewha Womans University Seoul Hospital, Ewha Womans University School of Medicine, Seoul, Korea

[3]Department of Physical and Rehabilitation Medicine, Center for Prevention and Rehabilitation, Heart Vascular Stroke Institute, Samsung Medical Center, Sungkyunkwan University School of Medicine, Seoul, Korea

4. Name and title of research drug/medical device managing pharmacist/device manager.

Not applicable.

5. The code name of the research drug or medical device, the general name of the main therapeutic chemical component (raw materials in the case of medical devices) and their quantity, formulation

(shape/structure in the case of medical devices), etc.

Not applicable.

6. Research sponsor name and address

Not applicable. This is an investigator initiated trial.

7. Background and purpose of the study

7.1 Background and rationale

Macular hole surgery and retinal detachmet surgery frequently involve intraocular tamponade. Intraocular tamponade is a method of injecting fillers into the eye during vitreoretinal surgery to use the buoyancy and provide force to press the retina and promote reattachment of retina and macular hole occlusion.[1] Facedown positioning, posture with the pupil facing the floor, lying on the stomach or sitting with the head bent deeply, is recommended after the surgery so that the buoyancy of the fillings in the eye works properly on macula. It is recommended to maintain the posture 3 to 10 days depending on the patient's condition.

Spending most of the time maintaining facedown posture, many patients experience musculoskeletal pain on their back neck, shoulder, and lower back. Patients with long-term posture restrictions in bed also complain of psychological isolation and boredom.[2-4] Maintaining facedown posture could be a more difficult experience than the surgery itself to patients and discomfort caused by prone posture may affect the outcome of retinal surgery by lowering patient compliance after surgery.[5]

Most of the vitreoretinal surgeons paid attention only to the outcome of the surgery, and the patient's discomfort caused by facedown posture was not greatly noticed previously. Currently, the intervention method for patient's pain complaint on musculoskeletal discomfort is limited. There has been no structured rehabilitation exercise proposed in the previous literature, and no study evaluated the effect of structured exercise therapy for facedown posture-related musculoskeletal pain.

Department of Ophthalmology and Department of physical and rehabilitation medicine, together, devised a novel structured exercise program for alleviation of musculoskeletal pain in back neck, shoulder, and lower back caused by facedown posture after retinal surgery. The investigators expect the rehabilitation exercise program to reduce musculoskeletal pain, increase subjective satisfaction after surgery, and maximize the effect of prone position (buoyancy pressing macula) by relieving posture related pain and

increasing posture maintenance time.

7.2 Study purpose

The objective of this study is to evaluate efficacy of the structured exercise program for reduction of musculoskeletal pain in patients who maintain a facedown posture with their pupils facing the floor after retinal surgery.

8. Target disease

- Retinal detachment (rhegmatogenous and tranctional)
- Macular hole

9. Selection criteria, exclusion criteria, target number of subjects and their basis

9.1 Subject selection criteria

(1) Patients 18 years of age or older who have been diagnosed with retinal detachment (rhegmatogenous retinal detachment, traction retinal detachment) or macular hole and who are scheduled for prone position after retinal surgery.

(2) Patients who signed the provided consent form.

9.2 Subject exclusion criteria

(1) Patients with pre-existing musculoskeletal disorder.

(2) patients with poor visiual acuity in the opposite eye (best-corrected visual acuity less than 20/200 by snellen chart) making patients hard to get the exercise training.

(3) Patients with posture prescriptions other than the prone position

9.3 Criteria for dropout

(1) Patients who stop rehabilitation exercise therapy in the middle.

(2) Patients whose exercise performance rate is significantly low. Patients who performed less than 2 times out of 3 times a day.

(3) Patients discharged from the hospital before the postoperative day 3 due to personal circumstances.

(4) Patients who use personal massage device.

9.4 Sample size calculation

No relevant studies have previously described the effect of structured exercise on FDP related pain. There has been only one study that evaluated FDP related pain quantitatively. A randomized controlled study performed by Adachi et al. reported the effects of aromatherapy massage on FDP related pain after vitrectomy.[6] Based on the clinical assumption that structured exercise program would have comparable or greater effect on FDP related pain, we conducted a sample size calculation using data from the previous report. We conducted a sample size calculation expecting an effect size of 0.9 based on post-treatment back pain score in aromatherapy group and control group of the previous study. The estimated sample size was 32 patients per group to detect differences between two groups at 90% power and a significance level of 0.05. Considering a 10 % loss to follow-up, we are aiming to recruit 35 patients in each arm.

9.5 Patients assignment and randomization

Eligible candidates will be invited to participate and to sign the consent form prior to surgery. After then, the block randomization method with block size of 4 or 6 will be implemented by an independent clinical trial consultant. Randomization will be performed using an online randomization service implemented by Sealed Envelope. Subjects will be allocated randomly 1:1 to either exercise group or control group based on pre-allocated codes placed in sealed opaque envelopes that were opened during the randomization step by a trial coordinator. Based on the code, each subject will be randomized to either the control group or the exercise group.

9.6 Masking

Due to the nature of the intervention, participants and researchers cannot be blinded to group allocation. The treating healthcare professionals (i.e. the Research Nurse and Clinical Research Fellow) will also not be masked due to nature of the service. The surgeon will be masked to allocation. The data manager and trial statistician will be masked to allocation throughout the study.

10. Study period

Total research period: IRB approval date ~ February 28, 2021

Patient recruitment period: IRB approval date - December 31, 2020

Statistical processing and result report writing period: 2 months from the date of completion of the last patient questionnaire

11. Research method

This study is for patients who are going to be prescribed facedown posture after retinal surgery.

Participating the study or not will not influence the clinical decision and surgical procedure, but only a pain assessment in the back neck, shoulder, and lower back will be additionally performed every day after the surgery for the study participants. Those who are assigned to the exercise group will additionally be instructed for a structured exercise program designed to keep the prone posture easily and to relieve musculoskeletal pain. The exercise group will be trained by an exercise therapist. All inpatient care and surgical procedures other than learning and performing rehabilitation exercise will be the same in both study groups.

Control group	Exercise group
After surgery, take and maintain facedown posture without getting additional training or exercise.	After surgery, subjects will be trained for a structured exercise routine by an exercise therapist and perform the exercise according to the instruction three times a day.
Pain assessment will be performed every day after surgery.	

All patients scheduled for vitreoretinal surgery for macular hole or retinal detachment is going to be admitted to the hospital the day before surgery (day 1). On the day 1, every patients will be evaluated for their eligibility, and those who are eligible candidates will be provided with information about the trial and invited to participate. They will be given enough time to consider their decision and the opportunity to ask questions. After informed consent is obtained, subjects will be randomized.

On the day of the retinal surgery, an exercise therapist who is affiliated to the Department of physical and rehabilitation medicine, Samsung Medical Center, will visits before surgery to educate the structured

rehabilitation exercise program. Details of a structured exercise program for the exercise group is as follow.

Rehabilitation exercise education consists of **(1) proper facedown positioning**, which would assist patients to maintain the prone position more easily and stably without giving too much stress to the body, and **(2) facedown exercise**, which would reduce the burden on muscle and joints induced by the facedown posture and enhance muscle strength to withstand the stress applied by the facedown posture.<br><br>**<Proper facedown positioning>**<br>-Lying on the bed, prone relaxed position<br>-Modified prone relaxation posture (head bowed position on the table, head bowed position on the bed)<br>-Standing and prone relaxed using a walker<br>**<Facedown Exercise>**<br>-Arms and Legs Exercises in a Bed Position<br>-Exercising in a four-legged position lying on the bed (back stretch)<br>-Walking in place from a prone position using a walker

After surgery, the pain score in numeric rating scale will be evaluated immediately after the surgery. The patients will perform the trained posture and exercise three times daily, and the exercise therapist will revisit on the postoperative day 1 to give feedback on posture and exercise. The pain score at the back neck, shoulder and lower back will be evaluated on the postoperative day 1, 2, and 3. There will be no difference in the treatment process other than using rehabilitation exercise therapy during the prone period. Patients will stay in the hospital until the postoperative day 3 and then be discharged. The trial will end on the day 5 (postoperative day 3) as the patients discharged from the hospital.

Overview of the schedule of the clinical trial.

	**Day 1**<br>**(Admission)**	**Day 2**<br>**(Surgery)**	**Day 3**<br>**(POD 1)**	**Day 4**<br>**(POD 2)**	**Day 5**<br>**(POD 3)**
Eligibility determination	X				
Informed consent	X				

Randomization	X				
Medical & Ophthalmic History	X				
Maintain facedown posture		X (after surgery)	X	X	X
Pain assessment		X (after surgery)	X	X	X
Exercise training by exercise therapist (with brochure and movie clip) for exercise group		X	X		
Three times of daily exercise for exercise group		X (after surgery)	X	X	X
Patient satisfaction questionnaire for exercise group					X

12. Clinical evaluation items and observation test method

Pre-operative data collection:

- Demographic data
- Laterality
- Comorbid systemic diseases (diabetes mellitus, arterial hypertension, etc.)
- Indication for vitrectomy
- previous history of ocular surgery
- combined cataract surgery
- methods of anesthesia

Post-operative data collection:

- Intraocular fillings (SF6, C3F8, Silicon oil)

- Operative time

- Pain scores measured by numeric rating scale at back neck, shoulder, and lower back

  - Pain scores will be measured on the surgery day and the postoperative day 1, 2, and 3.

- Patient satisfaction questionnaire for exercise group on the postoperative day 3.

13. Predicted side effects

In this study, there is no additional side effect to be predicted because the existing retinal surgery and prone position prescription proceed in the same manner except for the education and implementation of exercise therapy that can be combined with the prone position during hospitalization.

14. Outcome measures and statistical analysis

14.1. Primary outcome

Pain score measured by numeric rating scale at back neck, shoulder, and lower back on the postoperative day 3. Numeric rating scale is a score that measures pain. If there is no pain, it is marked as 0, and if the pain is the worst possible pain, it is measured as 10.

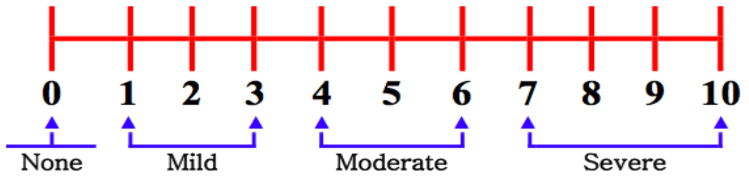

14.2. Secondary outcome measures

1) Pain score measured by numeric rating scale at back neck, shoulder, and lower back immediate after the surgery.

2) Pain score measured by numeric rating scale at back neck, shoulder, and lower back on the postoperative day 1.

3) Pain score measured by numeric rating scale at back neck, shoulder, and lower back on the postoperative day 2.

4) Difference between the study groups in pain score change over time at back neck, shoulder, and lower back

5) Patient's satisfaction on the structured exercise program.

The exercise group will be asked to answer the patients satisfaction questionnaire. The questionnaire items will be as follow.

(1) The rehabilitation program help patients recover health.

1: strongly disagree 2: disagree 3: neither agree nor disagree 4: agree 5: strongly disagree

(2) I want this exercise program the next time I have retinal surgery

1: strongly disagree 2: disagree 3: neither agree nor disagree 4: agree 5: strongly disagree

(3) I would recommend this program to other patients getting retinal surgery

1: strongly disagree 2: disagree 3: neither agree nor disagree 4: agree 5: strongly disagree

(4) Overall satisfaction

1: very disappointed 2: disappointed 3: neither satisfied nor disappointed 4: satisfied 5: very satisfied

14.3. Statistical analysis

Descriptive statistics for continuous variables will be presented in terms of mean, median, standard deviation, interquartile range, minimum and maximum values, and descriptive statistics for categorical variables are presented in terms of frequencies and ratios belonging to each category.

The comparison of continuous variables between the two groups will be tested with the two-sample t-test or Wilcoxon's rank sum test depending on whether the data are normal, and the comparison of categorical variables will be tested with the Chi-square test or Fisher's exact test. Since there will be three site for pain scoring, the statistical significance will be set at $p<0.017$ (0.05 divided by 3, Bonferroni's method) to control the inflation in the size of the type 1 error.

15. Evaluation criteria for safety including side effects, evaluation methods and reporting methods.

Not applicable

16. Patient Consent Form

Appendix 2

17. Rules for compensation for damages

-In case damages occur during participating the research, compensation will be made according to the in-hospital compensation standard.

18. Case report form

Appendix 3

19. Treatment standards after the study for trial participants

No influence

20. Protection of subject's personal information

The items will be record case report form after de-identifying the subject identification information, so that the subject's identity information is not exposed. In order to maintain the confidentiality of the subject, access to research data is restricted except for research directors and those who have been granted access from the principal investigator.

The study will be conducted after the IRB approval, complying with the Helsinki Declaration (revised in 2013) and ICH-GCP.

21. References

1. Kelly NE, Wendel RT. Vitreous surgery for idiopathic macular holes. Results of a pilot study. Arch Ophthalmol. 1991;109(5):654-659.

2. Harker R, McLaughlan R, MacDonald H, Waterman C, Waterman HA. Endless nights: Patients' experiences of posturing face-down following vitreoretinal surgery. Ophthalmic Nursing. 1996;6(2):11-15.

3. Brouzas D, Gourgounis N, Davou S, Loukianou E, Georgalas I, Koursandrea C. Ulnar neuropathy as a complication of retinal detachment surgery and face-down positioning. Case Rep Ophthalmol. 2011;2(2):243-245. doi:10.1159/000330692

4. Treister G, Wygnanski T. Pressure sore in a patient who underwent repair of a retinal tear with gas injection. Graefes Arch Clin Exp Ophthalmol. 1996;234(10):657-658. doi:10.1007/bf00185301

5. Seno Y, Shimada Y, Mizuguchi T, Tanikawa A, Horiguchi M. Compliance with the Face-down

Positioning after Vitrectomy and Gas Tamponade for Rhegmatogenous Retinal Detachments. Retina. 2015;35(7):1436-1440.

6. Adachi N, Munesada M, Yamada N, Suzuki H, Futohashi A, Shigeeda T, Kato S, Nishigaki M. Effects of aromatherapy massage on face-down posture-related pain after vitrectomy: a randomized controlled trial. Pain Manag Nurs. 2014 Jun;15(2):482-9. doi: 10.1016/j.pmn.2012.12.004. Epub 2013 Mar 5. PMID: 23466193.
