# Supplementary material for: A structured exercise to relieve musculoskeletal pain caused by face-down posture after retinal surgery: a randomized controlled trial
Source: Sci Rep. 2021 Nov 11;11:22074. doi: 10.1038/s41598-021-01182-w (PMC8586155; doi:10.1038/s41598-021-01182-w)
Supplement: Supplementary file 2 — Supplementary Information 2. [file 41598_2021_1182_MOESM2_ESM.pdf]

A Structured Exercise to Relieve Musculoskeletal Pain Caused by Face-down Posture after Retinal Surgery: A Randomized Controlled Trial

A Young Kim, MD[1,2,*]; Sungsoon Hwang, MD[1,3,*]; Se Woong Kang, MD, PhD[1]; So Yeon Shin, RN[4]; Won Hyuk Chang, MD, PhD[5]; Sang Jin Kim, MD, PhD[1]; and Hoon Noh, MD[1]

[*]A Young Kim and Sungsoon Hwang contributed equally to this work as co-first authors.

[1]Department of Ophthalmology, Samsung Medical Center, Sungkyunkwan University School of Medicine, Seoul, Republic of Korea

[2]Department of Ophthalmology, Ewha Womans University Seoul Hospital, Ewha Womans University School of Medicine, Seoul, Republic of Korea

[3]Department of Clinical Research Design and Evaluation, Samsung Advanced Institute for Health Sciences and Technology (SAIHST), Sungkyunkwan University, Seoul, Republic of Korea

[4]Department of Nursing, Samsung Medical Center, Seoul, Republic of Korea

[5]Department of Physical and Rehabilitation Medicine, Center for Prevention and Rehabilitation, Heart Vascular Stroke Institute, Samsung Medical Center, Sungkyunkwan University School of Medicine, Seoul, Republic of Korea

Corresponding Author:

Se Woong Kang, MD, PhD

Department of Ophthalmology, Samsung Medical Center, Sungkyunkwan University School of Medicine, #81 Irwon-ro, Gangnam-gu, Seoul 06351, Republic of Korea

Tel: +82-2-3410-3562, Fax: +82-2-3410-0074

Email: kangsewoong@gmail.com

SUPPLEMENTAL CONTENTS

Supplemental Document. Clinical trial protocol

Supplemental Figure 1. The detailed study design and the hospital course of the exercise group and control group.

Supplemental Figure 2. Satisfaction questionnaire items and patients' response to the questions.
